# Discrimination is associated with depression, anxiety, and loneliness symptoms among Asian and Pacific Islander adults during COVID-19 Pandemic

**DOI:** 10.1038/s41598-024-59543-0

**Published:** 2024-04-24

**Authors:** Cameron K. Ormiston, Paula D. Strassle, Eric Boyd, Faustine Williams

**Affiliations:** 1 Division of Intramural Research, National Institute on Minority Health and Health Disparities, 11545 Rockville Pike, Rockville, MD 20852 USA; 2 Department of Medical Education, Icahn School of Medicine at Mount Sinai, New York, NY USA; 3 Information Management Services, Inc., Calverton, MD USA

**Keywords:** Asian American, COVID-19 pandemic, Discrimination, Mental health, Pacific Islander, Medical research, Risk factors

## Abstract

In the United States, Asian and Pacific Islander (A/PI) communities have faced significant discrimination and stigma during the COVID-19 pandemic. We assessed the association between discrimination and depression, anxiety, and loneliness symptoms among Asian or Pacific Islander adults (n = 543) using data from a 116-item nationally distributed online survey of adults (≥ 18 years old) in the United States conducted between 5/2021–1/2022. Discrimination was assessed using the 5-item Everyday Discrimination Scale. Anxiety, depression, and loneliness symptoms were assessed using the 2-item Generalized Anxiety Disorder, 2-item Patient Health Questionnaire, and UCLA Loneliness Scale—Short form, respectively. We used multivariable logistic regression to estimate the association between discrimination and mental health. Overall, 42.7% of participants reported experiencing discrimination once a month or more. Compared with no discrimination, experiencing discrimination once a month was associated with increased odds of anxiety (Adjusted Odds Ratio [aOR] = 2.60, 95% CI = 1.38–4.77), depression (aOR = 2.58, 95% CI = 1.46–4.56), and loneliness (aOR = 2.86, 95% CI = 1.75–4.67). Experiencing discrimination once a week or more was associated with even higher odds of anxiety (aOR = 6.90, 95% CI = 3.71–12.83), depression, (aOR = 6.96, 95% CI = 3.80–12.74), and loneliness (aOR = 6.91, 95% CI = 3.38–13.00). Discrimination is detrimental to mental health, even at relatively low frequencies; however, more frequent discrimination was associated with worse mental health symptoms. Public health interventions and programs targeting anti-A/PI hate and reducing A/PI mental health burden are urgently needed.

## Introduction

Since the start of the COVID-19 pandemic, the United States (US) has seen a dramatic rise in anti-Asian and Pacific Islander (A/PI) discrimination^1,2^. Within the first month of the Asian Pacific Policy and Planning Council’s public reporting center for discrimination being active, they received 1,497 reports of anti-A/PI discrimination from across the US^3^. Since the start of the pandemic, anti-Asian hate crimes have increased by 344% and over half of Asian adults report that anti-Asian discrimination is more frequent compared to before the pandemic^1^. In one nationally representative survey of US adults, 1 in 3 Asian adults reported experiencing COVID-related discrimination, and Asian adults were more likely to experience discrimination compared to Black/African American, Hawaiian/Pacific Islander, Hispanic/Latino, multiracial, and White adults, even though COVID-related discrimination was common across all racial and ethnic minoritized groups^4^. Furthermore, almost a third of US adults believe China or Chinese Americans are to blame for the COVID-19 pandemic^5^.

Experiences of discrimination can have extensive, adverse impacts on the health, including mental health, of racial and ethnic minoritized individuals^2^. For example, after the September 11, 2001 attacks, the US saw a rise in discrimination, hate crimes, and negative attitudes towards Muslim Americans, which resulted in increased depressive and post-traumatic stress disorder (PTSD) symptoms among Muslim Americans^6–8^. Worse mental health due to discrimination after the 9/11 attacks was also reported among other minoritized groups, including Latino adults and Asian Americans^9,10^. In fact, national emergencies and crises, such as 9/11 or the COVID-19 pandemic, likely provide opportunities for white supremacy and privileged groups to reassert hegemony over the country’s sociopolitical and ideological environment thereby facilitating the exclusion of non-White communities^9^.

Discrimination experiences are said to trigger stress and trauma responses that can lead to chronic mental and physical health conditions such as cardiovascular disease, PTSD, and depression^1,6,11^. Indeed, numerous studies predating the pandemic have linked discrimination with depression, suicidal ideation, loneliness, and psychological distress among A/PI adults^1,12–14^. Persistent anti-Asian rhetoric in the US news, social media, and general population, and terms such as “Kung flu,” “Chinese Virus,” and other derogatory terms toward Asian communities during the pandemic will have deleterious, far-reaching effects on A/PI mental health^2,6,15^. For example, witnessing anti-Asian discrimination in public or seeing images of discrimination towards Asian individuals on the news or social media has been linked to depressive and anxiety symptoms among Asian adults during the pandemic^16^. And although A/PI adults were less likely to report having poor mental health compared to White adults prior to the pandemic^2,17–19^, recent research has found higher levels of mental health symptoms compared with White adults during the pandemic^2,20^.

Presently, our knowledge on the impact of discrimination during the COVID-19 pandemic on A/PI mental health is still developing. Although depression, anxiety, and loneliness have been examined among A/PI individuals before, most studies have focused on specific populations (e.g. adolescents, older adults), predate the COVID-19 pandemic, did not control for pre-existing mental health conditions, or did not utilize a national sample^1,6,21,22^. Understanding this relationship is important given the drastic increase in discrimination faced by A/PI communities in the US, and the already high risk of mental health concerns during the pandemic. Thus, the purpose of this analysis was to examine the association between discrimination and depression, anxiety, and loneliness symptoms during the COVID-19 pandemic in a national sample of A/PI adults living in the United States. We hypothesized higher frequency of discrimination would confer higher odds of depression, anxiety, and loneliness.

## Methods

### Study data and population

We conducted a comprehensive 116-item online survey that was nationally distributed throughout the US, which focused on mental health during the COVID-19 pandemic. Qualtrics LLC, which uses a national survey panel to conduct online surveys, distributed ten thousand surveys to adults (≥ 18 years old) living in the US from May 13, 2021, to January 9, 2022. Upon completing the survey, participants were given a $5–10 gift card from Qualtrics. As we were interested in assessing mental health during the pandemic among African, Asian, Hispanic/Latino, and Middle Eastern immigrant individuals, this group was oversampled during recruitment. Low-income (< $25,000 annual household income) and rural adults were also oversampled.

Initial survey responses received by Qualtrics (n = 5938, 59.4% response rate) were assessed via Expert Review Fraud Detection to prevent multiple submissions and ensure data integrity. Participants were removed from the final survey sample if they completed < 80% of the survey after accounting for skip pattern items or if they took < 5 min to complete the survey. Overall, 5,413 surveys were ultimately included in the final sample. For this study, we restricted our cohort to participants who self-identified as Asian and/or Pacific Islander (n = 534, 9.9% of sample). Informed consent was obtained from all individual participants included in the study.

The research protocol for the study was reviewed by the National Institutes of Health (NIH) Institutional Review Board (IRB) and was approved on December 23, 2020 (IRB#000308) as an exempt study. The NIH Intramural Research Program IRB Human Research Protections Program Office of Human Subjects Research Protections determined that our protocol did not involve human subjects and was excluded from IRB review. The study was performed in accordance with the ethical standards as laid down in the 1964 Declaration of Helsinki and its later amendments or comparable ethical standards. The data for the study is available upon request per the new Data Management and Sharing Agreement plan.

### Measures

An adapted version of the 5-item Everyday Discrimination Scale^23^ was used to assess for frequency of discrimination during the COVID-19 pandemic. Participants were asked, “Since the beginning of the Coronavirus/COVID-19 pandemic, how often have any of the following things happened to you? (1) You are treated with less courtesy or respect than other people; (2) You receive worse service than other people in restaurants or stores; (3) People act as though they think you are not intelligent; (4) People act as though they are afraid of you; and (5) You are threatened or assaulted”. For each scenario, participants had five answer options (Never, About once a month, About once a week, 2–3 times a week, and Daily or almost daily). Based on the responses, discrimination frequency was classified as having felt discrimination never, once a month, or once a week or more (About once a week/2–3 times a week/daily or almost daily). Participants were further asked to give the main reasons for their discrimination experiences. They could select all that apply from the following list of reasons: People think I have Coronavirus/COVID-19, Race, Ancestry or national origin, Immigration status, Gender, Age, Religion, Height, Weight, Sexual orientation, Education or income level, None of these or not applicable. The Everyday Discrimination Scale has been validated among a wide range of racial and ethnic groups, including Asian American adults^23–29^.

Anxiety, depression, and loneliness symptoms were assessed using the 2-item Generalized Anxiety Disorder (GAD-2)^30^, 2-item Patient Health Questionnaire (PHQ-2)^31^, and UCLA Loneliness 3-item Scale—Short form (ULS-3)^32^, respectively. The GAD-2 asks, “Over the last 2 weeks, how often have you been bothered by the following problems? (1) Feeling nervous, anxious, or on edge. (2) Not being able to stop or control worrying.” Respondents answered, Not at all (0), Several days (1), More than half the days (2), and Nearly every day (3) for each item. Response scores were summed, and a score of ≥ 3 indicated GAD (yes/present) and < 3 no/none^33^. The PHQ-2 asked, “Over the last 2 weeks, how often have you been bothered by the following problems? (1) Feeling nervous, anxious, or on edge. (2) Not being able to stop or control worrying”. Possible responses included Not at all (0), Several days (1), More than half the days (2), and Nearly every day (3) for each item. Response scores were summed, and a score of ≥ 3 indicated yes/present depression symptoms and < 3 no/none^31,34^. The ULS-3 asks participants, “How often do you feel that you lack companionship?”, “How often do you feel left out?”, “How often do you feel isolated from others?”. Response options included Hardly ever (1), Some of the time (2), and Often (3)^32^. Response scores were summed for each participant and a score of 3–5 = Not Lonely (no/none) and 6–9 = Lonely (yes/present)^32^. These scale cutoffs have been validated among a diverse range of populations^31–38^.

Other covariates were created from single questions from the questionnaire including age (18–44, 45–54, 55–64, ≥ 65 years old), country of birth (Born outside the US or Born in the US/US-born), income ($0–24,999; $25,000–34,999; $35,000–49,999; $50,000–74,999; ≥ $74,999), education (Less than high school, High school graduate, Technical or Some college, ≥ College degree), race (Asian or Pacific Islander), ethnicity (Hispanic/Latino or not Hispanic/Latino), sexual orientation (Bisexual, Else, Gay, Heterosexual, or Lesbian), gender identity (Man, Transgender and/or Non-binary, and Woman), marital status (Divorced/Separated, Married/Living with partner, Never married, Widowed), housing stability (Stable or Unstable) and employment (Employed [full or part-time] or Not employed).

### Statistical analyses

Prevalence of reasons for discrimination, anxiety, depression, and loneliness symptoms, overall and by frequency of COVID-related discrimination, were estimated using descriptive statistics. Multivariable logistic regression was used to estimate the association between discrimination (once a week or more vs. never; and monthly vs. never) and depression, anxiety, and loneliness symptoms, respectively. All models adjusted for race, ethnicity, age, gender identity, sexual orientation, income, country of birth, marital status, education, employment, housing stability, and history of mental health conditions (Anxiety disorder, Depressive disorder, Other mental health diagnosis). All analyses were performed using SAS version 9.4 (SAS Inc., Cary, NC) and SUDAAN Release 11.0.1 (Research Triangle Institute: Research Triangle Park, NC).

## Results

### Descriptive statistics of study sample

The majority of participants was Asian (91.9%), aged 18–44 years (61.0%), identified as woman (65.6%), heterosexual (89.5%), were employed (56.4%), and had a college degree or more (61.0%). Approximately 56.2% of the sample was born outside of the US. In terms of pre-existing mental health conditions, 10.7% had anxiety, 7.7% had depression, and 7.6% had a mental health diagnosis that was not anxiety or depression. See Table 1.

**Table 1:** Descriptive characteristics of study sample stratified by discrimination frequency.

	Overall n (%^a^)	Discrimination frequency			P-value
		Once a week or more n (%^b^)	About once a month n (%^b^)	Never n (%^b^)	
Total N (%)	534 (100)	105 (19.3)	127 (23.4)	311 (57.3)	
Race					0.0007
Asian	499 (91.9)	87 (17.4)	118 (23.7)	294 (58.9)	
Pacific Islander	44 (8.1)	18 (40.9)	9 (20.5)	17 (38.6)	
Age					< 0.0001
18–44 years old	331 (61.0)	82 (24.8)	89 (26.9)	160 (48.3)	
45–54 years old	79 (14.6)	12 (15.2)	18 (22.8)	49 (62.0)	
55–64 years old	62 (11.4)	9 (14.5)	12 (19.4)	41 (66.1)	
≥ 65 years old	71 (13.1)	2 (2.8)	8 (11.3)	61 (85.9)	
Gender identity					0.57
Man	177 (32.6)	35 (19.8)	41 (23.2)	101 (57.1)	
Transgender and/or non-binary	10 (1.8)	4 (40.0)	2 (20.0)	4 (40.0)	
Woman	356 (65.6)	66 (18.5)	84 (23.6)	206 (57.9)	
Sexual orientation					< 0.0001
Lesbian, gay, bisexual, else	57 (10.5)	23 (40.4)	12 (21.1)	22 (38.6)	
Heterosexual	486 (89.5)	82 (16.9)	115 (23.7)	289 (59.5)	
Annual household income					0.003
< $25,000	84 (15.5)	21 (25.0)	24 (28.6)	39 (46.4)	
$25,000–$34,999	61 (11.2)	17 (27.9)	15 (24.6)	29 (47.5)	
$35,000–$49,999	92 (16.9)	24 (26.1)	21 (22.8)	47 (51.1)	
$50,000–$74,999	92 (18.1)	21 (21.4)	23 (23.5)	54 (55.1)	
≥ $75,000	208 (38.3)	22 (10.6)	44 (21.2)	142 (68.3)	
Marital status					0.01
Divorced/separated	48 (8.8)	8 (16.7)	8 (16.7)	32 (66.7)	
Married/living with partner	343 (63.2)	56 (16.3)	81 (23.6)	206 (60.1)	
Never married	144 (26.5)	40 (27.8)	38 (26.4)	66 (45.8)	
Widowed	8 (1.5)	1 (12.5)	0 (0)	7 (87.5)	
Education					0.21
Less than high school	25 (4.6)	6 (24.0)	9 (36.0)	10 (40.0)	
High school graduate	72 (13.3)	15 (20.8)	17 (23.6)	40 (55.6)	
Technical or some college	115 (21.2)	29 (25.2)	27 (23.5)	59 (51.3)	
College degree or higher	331 (61.0)	55 (16.6)	74 (22.4)	202 (61.0)	
Employed	306 (56.4)	66 (21.6)	71 (23.2)	169 (55.2)	0.31
Place of birth					0.0003
Outside of United States	305 (56.2)	41 (13.4)	80 (26.2)	184 (60.3)	
United States/US-born	238 (43.8)	64 (26.9)	47 (19.8)	127 (53.4)	
History of mental health condition^1^					
Anxiety disorder	58 (10.7)	29 (50.0)	12 (20.7)	17 (29.3)	< 0.0001
Depressive disorder	42 (7.7)	20 (47.6)	6 (14.3)	16 (38.1)	< 0.0001
Other mental health diagnosis	41 (7.6)	25 (61.0)	6 (14.6)	10 (24.4)	< 0.0001
Unstable housing	37 (6.8)	17 (46.0)	9 (24.3)	11 (29.7)	< 0.0001

^a^Column percentages; ^b^Row percentages; ^1^Participants were able to select all that apply.

Overall, 42.7% of participants reported experiencing discrimination (once a month: 23.4%; once a week or more: 19.3%). Differences in discrimination frequency were seen by race, age, sexual orientation, annual household income, marital status, region of birth, history of mental health conditions (anxiety, depression, and other mental health diagnosis), and housing stability. See Table 1. When stratifying the overall sample by race, there were differences by sexual orientation, education, region of birth, history of mental health conditions (anxiety, depression, and other mental health diagnosis), and housing status. See Table 2.

**Table 2:** Descriptive characteristics of study sample stratified by race.

	Asian n (%^a^)	Pacific Islander n (%^a^)	P-value
Total N (%)	499 (100)	44 (100)	
Age			0.60
18–44 years old	302 (60.52)	29 (65.90)	
45–54 years old	73 (14.63)	6 (13.64)	
55–64 years old	56 (11.22)	6 (13.64)	
≥ 65 years old	68 (13.63)	3 (6.82)	
Gender identity			0.31
Man	165 (33.07)	12 (27.27)	
Transgender and/or non-binary	8 (1.60)	2 (4.55)	
Woman	326 (65.33)	30 (68.18)	
Sexual orientation			0.0002
Lesbian, gay, bisexual, else	45 (9.02)	12 (27.27)	
Heterosexual	454 (90.98)	32 (72.73)	
Annual household income			0.15
< $25,000	73 (14.63)	11 (25.00)	
$25,000–$34,999	54 (10.82)	7 (15.91)	
$35,000–$49,999	84 (16.83)	8 (18.18)	
$50,000–$74,999	90 (18.04)	8 (18.18)	
≥ $75,000	198 (39.68)	10 (22.72)	
Marital status			0.88
Divorced/separated	43 (8.62)	5 (11.36)	
Married/living with partner	317 (63.53)	26 (59.09)	
Never married	132 (26.45)	12 (27.27)	
Widowed	7 (1.40)	1 (2.27)	
Education			< 0.0001
Less than high school	21 (4.21)	4 (9.09)	
High school graduate	61 (12.22)	11 (25.00)	
Technical or some college	98 (19.64)	17 (38.64)	
College degree or higher	319 (63.93)	12 (27.27)	
Employed			0.10
No	223 (44.69)	14 (31.82)	
Yes	276 (55.31)	30 (68.18)	
Place of birth			< 0.0001
Outside of United States	295 (59.12)	10 (22.73)	
United States/US-born	204 (40.88)	34 (77.27)	
History of mental health condition^1^			
Anxiety disorder	47 (9.42)	11 (25.00)	0.001
Depressive disorder	31 (6.21)	11 (25.00)	< 0.0001
Other mental health diagnosis	32 (6.41)	9 (20.45)	0.0007
Unstable housing			0.06
No	468 (93.79)	38 (86.36)	
Yes	31 (6.21)	6 (13.64)	

^a^Column percentages; ^1^Participants were able to select all that apply.

**Figure 1:**
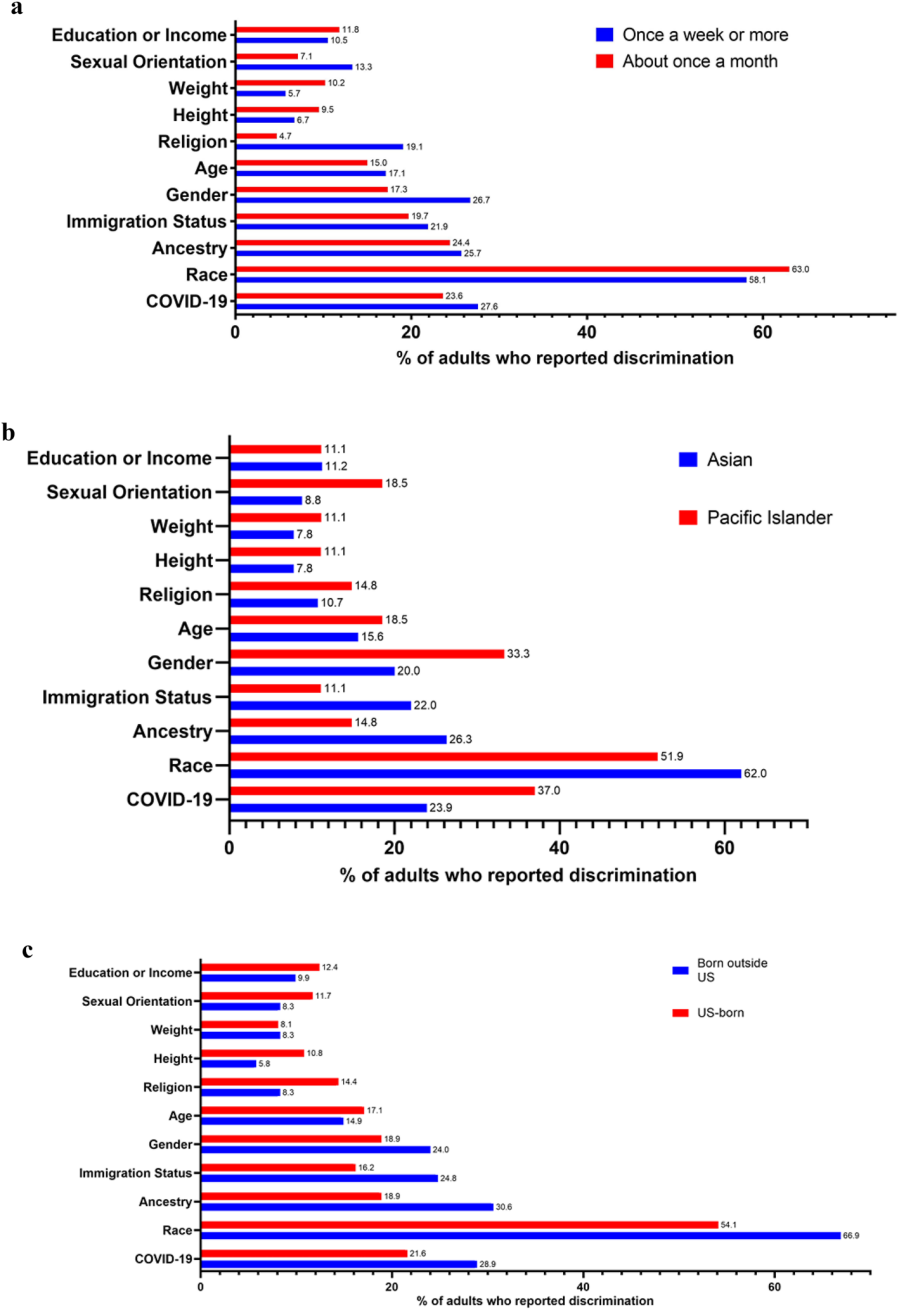
(**A**–**C**) Reasons for discrimination among individuals who reported experiencing discrimination during the pandemic (n = 232, 43.4%), stratified by (**A**) Discrimination frequency, (**B**) Race, and (**C**) Country of Birth. Participants were able to select more than one reason. (**A**) Reasons for discrimination stratified by discrimination frequency. (**B**) Reasons for discrimination stratified by race. (**C**) Reasons for discrimination stratified by place of birth.

Among participants who reported discrimination, 25.4% said the discrimination was COVID-19-related, 60.8% said it was due to race, 25.0% due to ancestry, 20.7% due immigration status, and 21.6% due to gender. See Supplemental Table 1. Participants who experienced discrimination more frequently were more likely to report the discrimination was due to religion (19.1% vs. 4.7%, p = 0.0006). See Supplemental Table 2 and Fig. 1A.

When stratifying participants who experienced discrimination by race, no differences were found between Asian and Pacific Islander respondents. See Supplemental Table 3 and Fig. 1B. After stratifying by place of birth (born outside US and US-born), people born outside US were more likely to report the discrimination was due to race (66.9% vs. 54.1%, p = 0.04) and ancestry (30.6% vs. 18.9%, p = 0.04). See Supplemental Table 4 and Fig. 1C.

**Figure 2:**
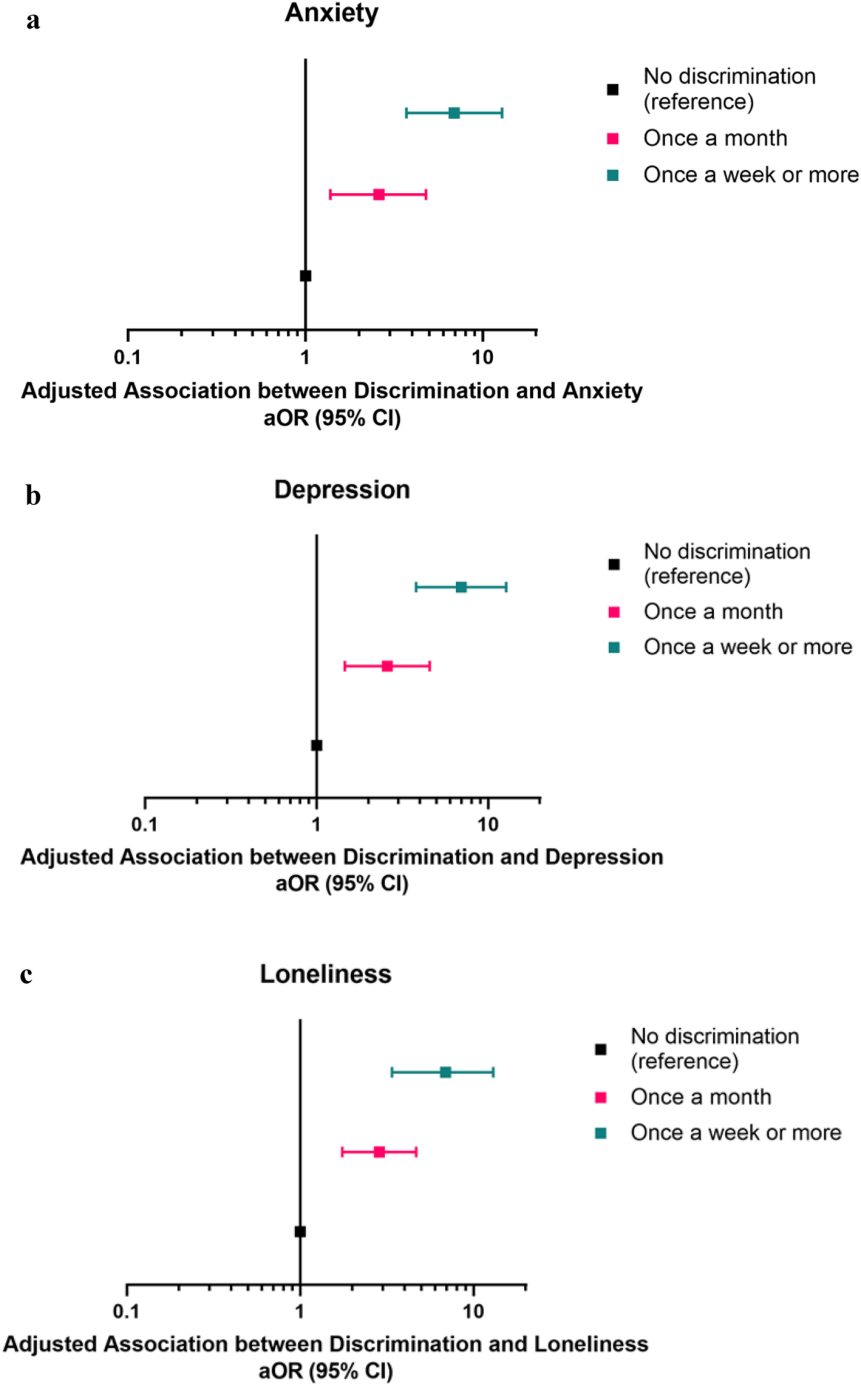
(**A**–**C**) Adjusted association between discrimination and (**A**) anxiety, (**B**) depression, and (**C**) loneliness symptoms among Asian and Pacific Islander adults. (**A**) Adjusted association between discrimination and anxiety. (**B**) Adjusted association between discrimination and depression. (**C**) Adjusted association between discrimination and depression and loneliness. All models were adjusted for race, ethnicity, age, gender identity, sexual orientation, income, country of birth, marital status, education, employment, housing stability, and history of mental health conditions (Anxiety disorder, Depressive disorder, Other mental health diagnosis). N = 499 Asian and Pacific Islander adults. See Supplemental Table 5 for the values of each aOR and 95% CI.

After adjusting for sociodemographic characteristics and history of mental health, we observed a dose–response between frequency of discrimination and increased odds of poor mental health. For example, compared with those reporting no discrimination, experiencing discrimination once a month was associated with almost three times the odds of anxiety (Adjusted Odds Ratio [aOR] = 2.60, 95% Confidence Interval [CI] = 1.38–4.77) and experiencing discrimination once a week or more was associated with over six times the odds of anxiety (aOR = 6.90, 95% CI = 3.71–12.83), Supplemental Table 5 and Fig. 2A. Similar trends were observed for both depression (once a month: aOR = 2.58, 95% CI = 1.46–4.56; once a week or more: aOR = 6.96, 95% CI = 3.80–12.74) and loneliness (once a month: aOR = 2.86, 95% CI = 1.75–4.67; aOR = 6.91, 95% CI = 3.38–13.00). See Supplemental Table 5 and Fig. 2B,C.

## Discussion

Using a national sample of US A/PI adults, we found that almost 50% reported experiencing discrimination, and among those who experienced discrimination, 25% reported that it was related to COVID-19. We also found that even when discrimination is experienced at relatively low frequencies (monthly), it had a substantial and detrimental impact on mental health; moreover, among individuals who experienced discrimination more frequently (once a week or more) the odds of poor mental health was even greater. Overall, this study represents one of the most recent and comprehensive assessments of the impact of discrimination on mental health in the US A/PI community during the COVID-19 pandemic.

Since the start of the pandemic, depressive symptoms among US adults and global anxiety symptoms have both tripled, and global loneliness symptoms have significantly increased^39–41^. These trends may be due to a multitude of reasons, including diseases-related anxiety, isolation due to quarantine and stay-at-home orders, and stress from economic and financial instability^41^. A/PI individuals, however, may be doubly burdened during the pandemic, experiencing fear, stress, and isolation due to not only the pandemic, but also due to anti-Asian discrimination, stigmatization, and violence^2,6^.

The present study found the total prevalence of discrimination among A/PI, Asian, and Pacific Islander adults to be 42.7%, 41.1%, and 61.4%, respectively. These findings are comparable with existing research. For example, a study utilizing COVID-19 Effects on the Mental and Physical Health of Asian Americans and Pacific Islanders Survey Study data found 60.7% of A/PI adults reported discrimination^42^. An online survey of Asian adults in Florida found 56.5% experienced discrimination during the pandemic^6^. Other studies report the prevalence of COVID-19-related discrimination to be 20–67% among Asian adults^1,2,4,43^. Among Pacific Islander adults, the prevalence of discrimination during the pandemic is estimated to be 22.8–40.5%^4,42^. Research prior to the pandemic show the prevalence of discrimination among Asian and Pacific Islander adults living in the US was 13–50% and 48–52%, respectively^13,42^. Both the present study and prior studies therefore highlight the increase in discrimination among A/PI adults and an urgent need to address this issue given discrimination’s harmful effects on mental health.

Prior research has also shown anti-Asian discrimination during the pandemic has negative effects on mental health^2,43^ and may have led to the Asian-White mental health gap now seen in the US^2^. Our findings also mirror existing research on the link between discrimination and A/PI mental health both prior to and during the pandemic^1,2,16,43–46^. A recent study on Asian/Asian American young adults found COVID-19-related discrimination to be significantly associated with PTSD symptoms after controlling for demographics, socioeconomic status, lifetime discrimination, and pre-existing mental health conditions^1^. Furthermore, an analysis of a national survey of 245 Asian/Asian American adults found discrimination during the pandemic was significantly associated with depressive symptoms as assessed using the 20-item Center for Epidemiologic Studies of Depression Scale^43^. Given A/PI adults report lower rates of using mental health services and discrimination has been previously associated with lower mental health service utilization among Asian adults, providing community-based, accessible, anti-racist, and culturally competent services is increasingly important^2,3,12^.

There are a number of limitations to consider for our study. First, our study is cross-sectional, meaning we cannot infer any directionality or causality of our findings. While we assume discrimination leads to mental health symptoms, individuals with mental health conditions experience significant barriers and stigmatization in society and report high levels of discrimination due to their mental health status, particularly those of racial and ethnic minoritized groups^47–50^. Second, given small sample sizes, we aggregated the Asian and Pacific Islander samples, and were unable to perform Asian and Pacific Islander analyses separately and among subgroups. Future research should aim disaggregate data and examine the link between discrimination and mental health outcomes across Asian and Pacific Islander subgroups and other intersectional identities (e.g., generational immigration status). The heterogenous experiences between these groups therefore may not be captured in our results. Additionally, the sample size for many of our cell counts were small, which may also introduce power issues that can impact the results and are likely to be unstable in adjusted models. Fourth, this was a convenience sample, which limits statistical inference, replication, and generalization of the results. Data integrity may also be a concern of convenience sampling; however we had several safeguards in place to prevent this issue. Fourth, the survey was conducted in English, thus individuals with limited English proficiency may have been underrepresented. Finally, the survey was online and individuals with limited access to the internet may not have been captured in the results.

## Conclusions

Among a national sample of A/PI adults, discrimination was associated with anxiety, depression, and loneliness symptoms. Although odds of mental health symptoms increased with increased frequency of discrimination, our results highlight the deleterious impact of discrimination even at ‘low’ levels of frequency. The pandemic and discrimination will likely have far-reaching, sustained impacts on A/PI mental and physical health. As such, health practitioners need to be educated on the unique experiences of A/PI adults, prepared to effectively screen for and treat these issues, and utilize their unique positions as leaders in health and society to stand up to racism and discrimination. Interventions that both target anti-A/PI hate and disinformation and address the growing mental health burden among A/PI in the US will be essential to mitigating potential long-term, negative effects of the pandemic among A/PI communities.

### Supplementary Information

Supplementary Tables.

## Data Availability

The data are available by making a request through Dr. FW per the new Data Management and Sharing Agreement plan.
