# Supplementary material for: Discrimination is associated with depression, anxiety, and loneliness symptoms among Asian and Pacific Islander adults during COVID-19 Pandemic
Source: Sci Rep. 2024 Apr 24;14:9417. doi: 10.1038/s41598-024-59543-0 (PMC11043456; doi:10.1038/s41598-024-59543-0)
Supplement: Supplementary file 1 — Supplementary Tables. [file 41598_2024_59543_MOESM1_ESM.docx]

**Tables**

**Table 1.** Descriptive characteristics of study sample stratified by discrimination frequency

|  |  | **Discrimination Frequency** | | |  |
| --- | --- | --- | --- | --- | --- |
|  | **Overall n (%^a^)** | **Once a week or more n (%^b^)** | **About once a month n (%^b^)** | **Never n (%^b^)** | **P-value** |
| **Total N (%)** | 534 (100) | 105 (19.3) | 127 (23.4) | 311 (57.3) |  |
| **Race** |  |  |  |  | 0.0007 |
| Asian | 499 (91.9) | 87 (17.4) | 118 (23.7) | 294 (58.9) |  |
| Pacific Islander | 44 (8.1) | 18 (40.9) | 9 (20.5) | 17 (38.6) |  |
| **Age** |  |  |  |  | <0.0001 |
| 18-44 years old | 331 (61.0) | 82 (24.8) | 89 (26.9) | 160 (48.3) |  |
| 45-54 years old | 79 (14.6) | 12 (15.2) | 18 (22.8) | 49 (62.0) |  |
| 55-64 years old | 62 (11.4) | 9 (14.5) | 12 (19.4) | 41 (66.1) |  |
| ≥65 years old | 71 (13.1) | 2 (2.8) | 8 (11.3) | 61 (85.9) |  |
| **Gender Identity** |  |  |  |  | 0.57 |
| Man | 177 (32.6) | 35 (19.8) | 41 (23.2) | 101 (57.1) |  |
| Transgender and/or Non-binary | 10 (1.8) | 4 (40.0) | 2 (20.0) | 4 (40.0) |  |
| Woman | 356 (65.6) | 66 (18.5) | 84 (23.6) | 206 (57.9) |  |
| **Sexual Orientation** |  |  |  |  | <0.0001 |
| Lesbian, Gay, Bisexual, Else | 57 (10.5) | 23 (40.4) | 12 (21.1) | 22 (38.6) |  |
| Heterosexual | 486 (89.5) | 82 (16.9) | 115 (23.7) | 289 (59.5) |  |
| **Annual Household Income** |  |  |  |  | 0.003 |
| <$25,000 | 84 (15.5) | 21 (25.0) | 24 (28.6) | 39 (46.4) |  |
| $25,000-$34,999 | 61 (11.2) | 17 (27.9) | 15 (24.6) | 29 (47.5) |  |
| $35,000-$49,999 | 92 (16.9) | 24 (26.1) | 21 (22.8) | 47 (51.1) |  |
| $50,000-$74,999 | 92 (18.1) | 21 (21.4) | 23 (23.5) | 54 (55.1) |  |
| ≥$75,000 | 208 (38.3) | 22 (10.6) | 44 (21.2) | 142 (68.3) |  |
| **Marital Status** |  |  |  |  | 0.01 |
| Divorced/Separated | 48 (8.8) | 8 (16.7) | 8 (16.7) | 32 (66.7) |  |
| Married/Living with partner | 343 (63.2) | 56 (16.3) | 81 (23.6) | 206 (60.1) |  |
| Never married | 144 (26.5) | 40 (27.8) | 38 (26.4) | 66 (45.8) |  |
| Widowed | 8 (1.5) | 1 (12.5) | 0 (0) | 7 (87.5) |  |
| **Education** |  |  |  |  | 0.21 |
| Less than high school | 25 (4.6) | 6 (24.0) | 9 (36.0) | 10 (40.0) |  |
| High school graduate | 72 (13.3) | 15 (20.8) | 17 (23.6) | 40 (55.6) |  |
| Technical or Some college | 115 (21.2) | 29 (25.2) | 27 (23.5) | 59 (51.3) |  |
| College degree or Higher | 331 (61.0) | 55 (16.6) | 74 (22.4) | 202 (61.0) |  |
| **Employed** | 306 (56.4) | 66 (21.6) | 71 (23.2) | 169 (55.2) | 0.31 |
| **Place of birth** |  |  |  |  | 0.0003 |
| Outside of United States | 305 (56.2) | 41 (13.4) | 80 (26.2) | 184 (60.3) |  |
| United States/US-born | 238 (43.8) | 64 (26.9) | 47 (19.8) | 127 (53.4) |  |
| **History of mental health condition^1^** |  |  |  |  |  |
| Anxiety disorder | 58 (10.7) | 29 (50.0) | 12 (20.7) | 17 (29.3) | <0.0001 |
| Depressive disorder | 42 (7.7) | 20 (47.6) | 6 (14.3) | 16 (38.1) | <0.0001 |
| Other mental health diagnosis | 41 (7.6) | 25 (61.0) | 6 (14.6) | 10 (24.4) | <0.0001 |
| **Unstable housing** | 37 (6.8) | 17 (46.0) | 9 (24.3) | 11 (29.7) | <0.0001 |

^a^Column percentages; ^b^Row percentages; ^1^Participants were able to select all that apply.

**Table 2.** Descriptive characteristics of study sample stratified by race

|  | **Asian n (%^a^)** | **Pacific Islander**  **n (%^a^)** | **P-value** |
| --- | --- | --- | --- |
| **Total** | 499 | 44 |  |
| **Age** |  |  | 0.60 |
| 18-44 years old | 302 (60.52) | 29 (65.91) |  |
| 45-54 years old | 73 (14.63) | 6 (13.64) |  |
| 55-64 years old | 56 (11.22) | 6 (13.64) |  |
| ≥65 years old | 68 (13.63) | 3 (6.82) |  |
| **Gender Identity** |  |  | 0.31 |
| Man | 165 (33.07) | 12 (27.27) |  |
| Transgender and/or Non-binary | 8 (1.60) | 2 (4.55) |  |
| Woman | 326 (65.33) | 30 (68.18) |  |
| **Sexual Orientation** |  |  | 0.0002 |
| Lesbian, Gay, Bisexual, Else | 45 (9.02) | 12 (27.27) |  |
| Heterosexual | 454 (90.98) | 32 (72.73) |  |
| **Annual Household Income** |  |  | 0.15 |
| <$25,000 | 73 (14.63) | 11 (25.00) |  |
| $25,000-$34,999 | 54 (10.82) | 7 (15.91) |  |
| $35,000-$49,999 | 84 (16.83) | 8 (18.18) |  |
| $50,000-$74,999 | 90 (18.04) | 8 (18.18) |  |
| ≥$75,000 | 198 (39.68) | 10 (22.72) |  |
| **Marital Status** |  |  | 0.88 |
| Divorced/Separated | 43 (8.62) | 5 (11.36) |  |
| Married/Living with partner | 317 (63.53) | 26 (59.09) |  |
| Never married | 132 (26.45) | 12 (27.27) |  |
| Widowed | 7 (1.40) | 1 (2.27) |  |
| **Education** |  |  | <0.0001 |
| Less than high school | 21 (4.21) | 4 (9.09) |  |
| High school graduate | 61 (12.22) | 11 (25.00) |  |
| Technical or Some college | 98 (19.64) | 17 (38.64) |  |
| College degree or Higher | 319 (63.93) | 12 (27.27) |  |
| **Employed** |  |  | 0.10 |
| No | 223 (44.69) | 14 (31.82) |  |
| Yes | 276 (55.31) | 30 (68.18) |  |
| **Place of birth** |  |  | <0.0001 |
| Outside of United States | 295 (59.12) | 10 (22.73) |  |
| United States/US-born | 204 (40.88) | 34 (77.27) |  |
| **History of mental health condition^1^** |  |  |  |
| Anxiety disorder | 47 (9.42) | 11 (25.00) | 0.001 |
| Depressive disorder | 31 (6.21) | 11 (25.00) | <0.0001 |
| Other mental health diagnosis | 32 (6.41) | 9 (20.45) | 0.0007 |
| **Unstable housing** |  |  | 0.06 |
| No | 468 (93.79) | 38 (86.36) |  |
| Yes | 31 (6.21) | 6 (13.64) |  |

^a^Column percentages; ^1^Participants were able to select all that apply

**Supplemental Tables**

**Supplemental Table 1**. Reasons for discrimination

| **Reasons for discrimination^a^** | **n (%)** |
| --- | --- |
| COVID-19 | 59 (25.4) |
| Race | 141 (60.8) |
| Ancestry | 58 (25.0) |
| Immigration status | 48 (20.7) |
| Gender | 50 (21.6) |
| Age | 50 (21.6) |
| Religion | 37 (16.0) |
| Height | 26 (11.2) |
| Weight | 19 (8.2) |
| Sexual orientation | 19 (8.2) |
| Education or Income | 23 (11.2) |

^a^Participants were able to select all that apply

**Supplemental Table 2.** Reasons for discrimination stratified by frequency of discrimination

| **Reasons for discrimination^a^** | **Once a week or more**  **n (%)** | **About once a month n (%)** | **P-value** |
| --- | --- | --- | --- |
| COVID-19 | 29 (27.6) | 30 (23.6) | 0.49 |
| Race | 61 (58.1) | 80 (63.0) | o.45 |
| Ancestry | 27 (25.7) | 31 (24.4) | 0.82 |
| Immigration status | 23 (21.9) | 25 (19.7) | 0.68 |
| Gender | 28 (26.7) | 22 (17.3) | 0.08 |
| Age | 18 (17.1) | 19 (15.0) | 0.65 |
| Religion | 20 (19.1) | 6 (4.7) | 0.0006 |
| Height | 7 (6.7) | 12 (9.5) | 0.44 |
| Weight | 6 (5.7) | 13 (10.2) | 0.21 |
| Sexual orientation | 14 (13.3) | 9 (7.1) | 0.11 |
| Education or Income | 11 (10.5) | 15 (11.8) | 0.75 |

^a^Participants were able to select all that apply

**Supplemental Table 3.** Reasons for discrimination stratified by race

| **Reasons for discrimination^a^** | **Asian n (%)** | **Pacific Islander n (%)** | **P-value** |
| --- | --- | --- | --- |
| COVID-19 | 49 (23.9) | 10 (37.0) | 0.14 |
| Race | 127 (62.0) | 14 (51.9) | 0.31 |
| Ancestry | 54 (26.3) | 4 (14.8) | 0.19 |
| Immigration status | 45 (22.0) | 3 (11.1) | 0.19 |
| Gender | 41 (20.0) | 9 (33.3) | 0.11 |
| Age | 32 (15.6) | 5 (18.5) | 0.70 |
| Religion | 22 (10.7) | 4 (14.8) | 0.53 |
| COVID-19 | 49 (23.9) | 10 (37.0) | 0.14 |
| Height | 16 (7.8) | 3 (11.1) | 0.56 |
| Weight | 16 (7.8) | 3 (11.1) | 0.56 |
| Sexual orientation | 18 (8.8) | 5 (18.5) | 0.11 |
| Education or Income | 23. (11.2) | 3 (11.1) | 0.99 |

^a^Participants were able to select all that apply

**Supplemental Table 4.** Reasons for discrimination stratified by place of birth

| **Reasons for discrimination^a^** | **Born outside US n (%)** | **US-born n (%)** | **P-value** |
| --- | --- | --- | --- |
| COVID-19 | 35 (28.9) | 24 (21.6) | 0.20 |
| Race | 81 (66.9) | 60 (54.1) | 0.04 |
| Ancestry | 37 (30.6) | 21 (18.9) | 0.04 |
| Immigration status | 30 (24.8) | 18 (16.2) | 0.11 |
| Gender | 29 (24.0) | 21 (18.9) | 0.35 |
| Age | 18 (14.9) | 19 (17.1) | 0.64 |
| Religion | 10 (8.3) | 16 (14.4) | 0.14 |
| Height | 7 (5.8) | 12 (10.8) | 0.16 |
| Weight | 10 (8.3) | 9 (8.1) | 0.97 |
| Sexual orientation | 10 (8.3) | 13 (11.7) | 0.38 |
| Education or Income | 11 (9.9) | 15 (12.4) | 0.55 |

^a^Participants were able to select all that apply

**Supplemental Table 5.** Logistic regression results for association between discrimination and

mental health symptoms (anxiety, depression, and loneliness)

| **Frequency of discrimination** | **Depression**  **AOR (95% CI)** | **Anxiety**  **AOR (95% CI)** | **Loneliness**  **AOR (95% CI)** |
| --- | --- | --- | --- |
| None | Ref | Ref | Ref |
| Once a month | 2.58 (1.46-4.56) | 2.60 (1.38-4.77) | 2.86 (1.75-4.67) |
| Once a week or more | 6.96 (3.80-12.74 | 6.90 (3.71-12.83) | 6.91 (3.38-13.00) |

Adjusted for race, ethnicity, age, gender identity, sexual orientation, income, country of birth,

marital status, education, employment, housing stability, and history of mental health conditions

(Anxiety disorder, Depressive disorder, Other mental health diagnosis). AOR= Adjusted odds

ratio, 95% CI= 95% confidence interval.

**Figures**

**Figures 1A-C.** Reasons for discrimination among individuals who reported experiencing discrimination during the pandemic (n=232, 43.4%), stratified by (A) Discrimination frequency, (B) Race, and (C) Country of Birth. Participants were able to select more than one reason.

**Figure 1A**. Reasons for discrimination stratified by discrimination frequency


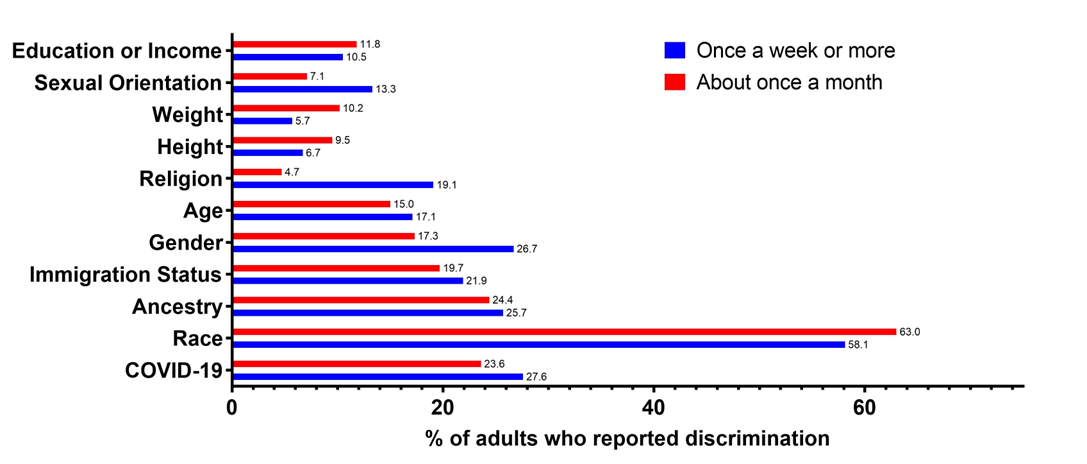


**Figure 1B**. Reasons for discrimination stratified by race


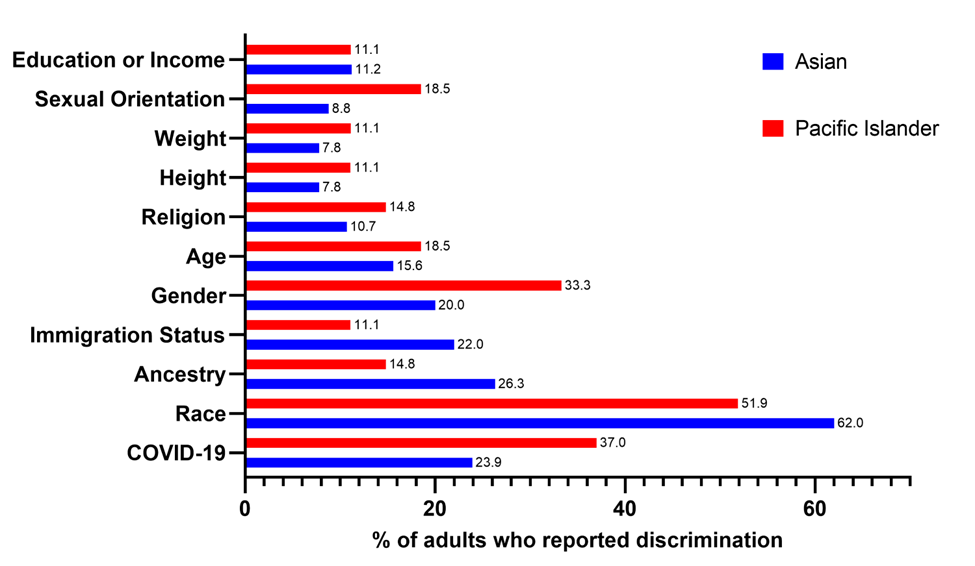


**Figure 1C**. Reasons for discrimination stratified by place of birth


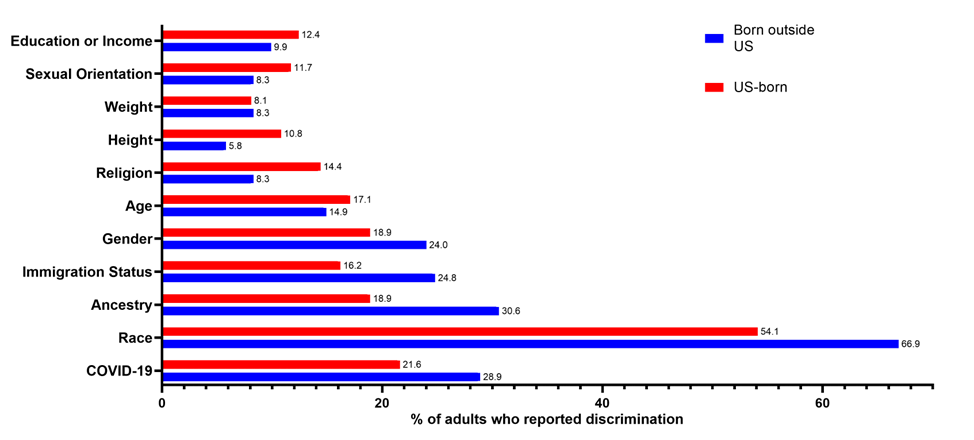


**Figures 2A-C.** Adjusted association between discrimination and A) anxiety, B) depression, and C) loneliness symptoms among Asian and Pacific Islander adults.

**Figure 2A.** Adjusted association between discrimination and anxiety
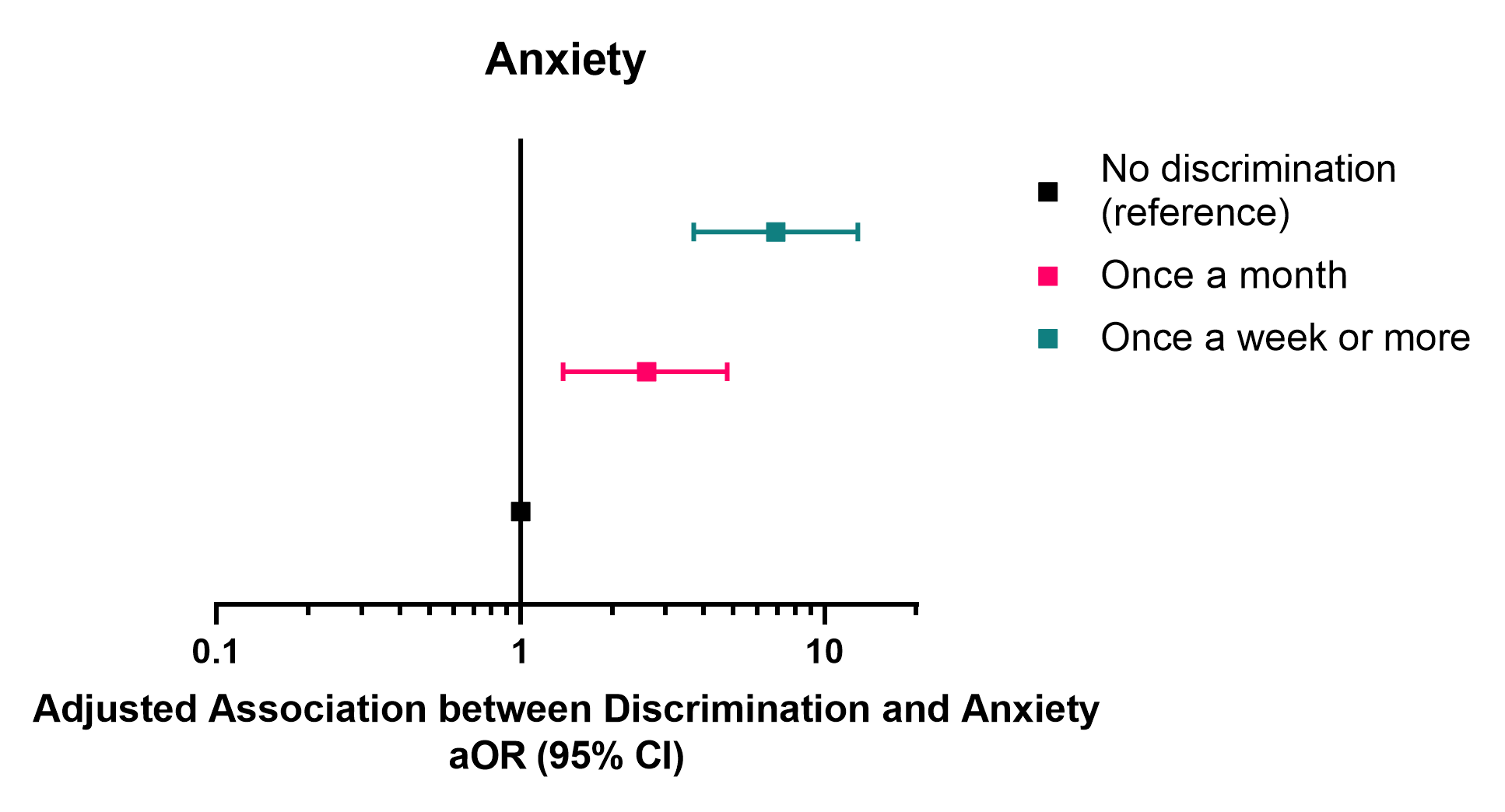


**Figure 2B.** Adjusted association between discrimination and depression

**
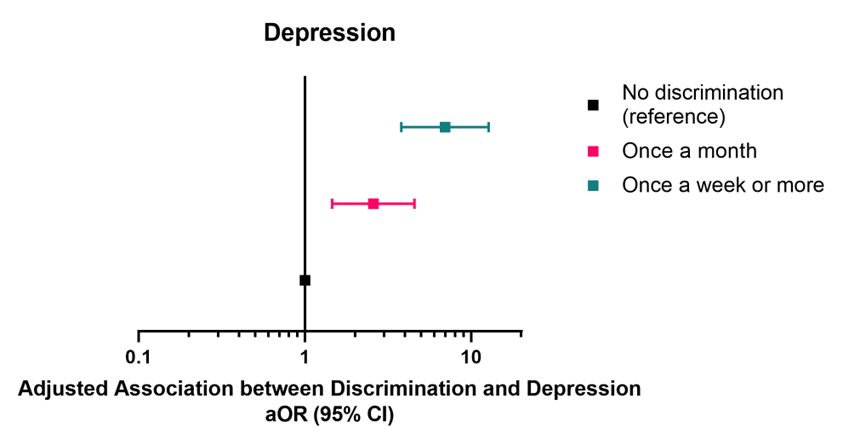
**

**Figure 2C.** Adjusted association between discrimination and depression and loneliness

**
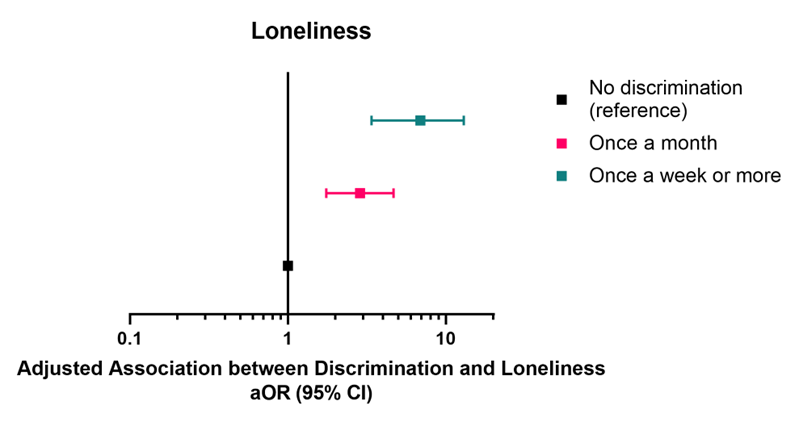
**

**Figures 2A-C.** All models were adjusted for race, ethnicity, age, gender identity, sexual orientation, income, country of birth, marital status, education, employment, housing stability, and history of mental health conditions (Anxiety disorder, Depressive disorder, Other mental health diagnosis). N=499 Asian and Pacific Islander adults. See Supplemental Table 5 for the values of each aOR and 95% CI.
